# Controlled Hypoxia Acutely Prevents Physical Inactivity-Induced Peripheral BDNF Decline

**DOI:** 10.3390/ijms25147536

**Published:** 2024-07-09

**Authors:** Yves Duderstadt, Stefanie Schreiber, Johannes Burtscher, Lutz Schega, Notger G. Müller, Tanja Brigadski, Rüdiger C. Braun-Dullaeus, Volkmar Leßmann, Patrick Müller

**Affiliations:** 1 Division of Cardiology and Angiology, University Hospital Magdeburg, 39120 Magdeburg, Germany; yves.duderstadt@med.ovgu.de (Y.D.);; 2 German Center for Neurodegenerative Diseases (DZNE), 39120 Magdeburg, Germany; 3 Department of Sports Science, Chair of Health and Physical Activity, Otto-von-Guericke University, 39104 Magdeburg, Germany; 4 Center for Intervention and Research on Adaptive and Maladaptive Brain Circuits Underlying Mental Health (C-I-R-C), 39120 Magdeburg, Germany; 5 Division of Neurology, University Hospital Magdeburg, 39120 Magdeburg, Germany; 6 Center for Behavioral Brain Sciences (CBBS), 39120 Magdeburg, Germany; 7 German Center for Mental Health (DZPG), 39120 Magdeburg, Germany; 8 Institute of Sports Science, University Innsbruck, 6020 Innsbruck, Austria; johannes.burtscher@uibk.ac.at; 9 Faculty of Health Sciences Brandenburg, University of Potsdam, 14476 Potsdam, Germany; 10 Institute of Physiology, Otto-von-Guericke University, 39120 Magdeburg, Germany; 11 Department of Informatics and Microsystems Technology, University of Applied Sciences, 67659 Kaiserslautern, Germany

**Keywords:** BDNF, hypoxia, physical inactivity, cognition

## Abstract

Brain-derived neurotrophic factor (BDNF) is a crucial mediator of neuronal plasticity. Here, we investigated the effects of controlled normobaric hypoxia (NH) combined with physical inactivity on BDNF blood levels and executive functions. A total of 25 healthy adults (25.8 ± 3.3 years, 15 female) were analyzed in a randomized controlled cross-over study. Each intervention began with a 30 min resting phase under normoxia (NOR), followed by a 90 min continuation of NOR or NH (peripheral oxygen saturation [SpO_2_] 85–80%). Serum and plasma samples were collected every 15 min. Heart rate and SpO_2_ were continuously measured. Before and after each exposure, cognitive tests were performed and after 24 h another follow-up blood sample was taken. NH decreased SpO_2_ (*p* < 0.001, η_p_^2^ = 0.747) and increased heart rate (*p* = 0.006, η_p_^2^ = 0.116) significantly. The 30-min resting phase under NOR led to a significant BDNF reduction in serum (*p* < 0.001, η_p_^2^ = 0.581) and plasma (*p* < 0.001, η_p_^2^ = 0.362). Continuation of NOR further significantly reduced BDNF after another 45 min (*p* = 0.018) in serum and after 30 min (*p* = 0.040) and 90 min (*p* = 0.005) in plasma. There was no significant BDNF decline under NH. A 24 h follow-up examination showed a significant decline in serum BDNF, both after NH and NOR. Our results show that NH has the potential to counteract physical inactivity-induced BDNF decline. Therefore, our study emphasizes the need for a physically active lifestyle and its positive effects on BDNF. This study also demonstrates the need for a standardized protocol for future studies to determine BDNF in serum and plasma.

## 1. Introduction

Understanding the molecular mechanisms of learning and memory is a fundamental prerequisite for positively influencing cognitive vitality across the lifespan. An essential modulator of cognitive performance and flexibility is the neurotrophin brain-derived neurotrophic factor (BDNF). The presence of BDNF has already been demonstrated in various regions of the central nervous system (CNS), with the strongest synthesis and expression occurring in the human hippocampus and the cerebral cortex [1]. BDNF is well associated with neuronal survival, synaptic plasticity, neuronal development and differentiation [1,2,3,4,5]. BDNF is also synthesized and secreted from cells outside of the central nervous system, e.g., in motoneurons and Schwann cells [6]. Even in tissues outside the nervous system, BDNF is synthesized [5] and modulates cellular functions, such as influencing the survival of endothelial cells, and angiogenesis [7].

As we age, there is an individual physiological decline in BDNF [8,9,10]. In addition, neurodegenerative diseases such as Alzheimer’s disease, Parkinson’s disease and depression are associated with a reduction in BDNF [9,11,12,13]. On the other hand, numerous studies have shown that increased BDNF positively correlates with cognitive abilities, especially memory performances [8,14,15]. Up to date, several factors (e.g., physical activity, nutrition, sleep quality, chronic stress) are known to influence BDNF levels [2,16,17,18,19]. In previous studies, our group has shown positive effects of exercise, especially sportive dancing, on BDNF levels in elderly people [20,21]. Additionally, previous studies demonstrated potential positive effects of controlled hypoxia on BDNF [22,23,24,25]. In general, the benefits and detriments of hypoxia are determined by the severity, frequency, and duration of hypoxia exposures [26]. Controlled hypoxia has the potential to trigger various adaptive physiological metabolic processes [26,27,28,29,30] and exert numerous positive health effects similar to aerobic physical activity [31]. In sports medicine, continuous and intermittent hypoxic training is widely used to increase human physical performance, e.g., through increased erythropoiesis or angiogenesis [32,33]. An insufficient supply of O_2_ leads to a decrease in the O_2_ saturation of the blood (hypoxemia). This is also defined as arterial oxygen partial pressure (paO_2_) of ≤60 mmHg. A reduced paO_2_ value in the blood in turn leads to lower oxygen in organic tissues (hypoxia). Similarly, there is evidence that controlled hypoxia may be neuroprotective [34,35,36]. However, the underlying neurobiological mechanisms are poorly understood.

Regarding the possible effects of controlled hypoxia on BDNF, research so far has provided inconsistent results [22,23,24,25,37,38,39]. However, investigating the effects of daily intermittent normobaric hypoxia over two weeks in young adults in a pilot study, we found reduced BDNF plasma levels [37].

The aim of this study was to determine the effect of passive NH and NOR exposure on the time course of BDNF levels and executive functions in young healthy participants. Currently, there are no data available regarding possible effects of a passive resting phase on BDNF. Tarassova and colleagues performed a 15 min resting phase before BDNF determination [40]. However, the first blood sample in their study was taken after the resting phase. Since approximately 99% of peripheral BDNF is stored in platelets and only free circulating BDNF is measurable in blood plasma, both serum and plasma levels of BDNF were examined in this study [41,42]. Additionally, we investigated whether there were alterations in BDNF levels 24 h post intervention and if the type of blood collection material has an influence on measured BDNF levels.

## 2. Results

### 2.1. Physiological Parameters

**Figure 1:**
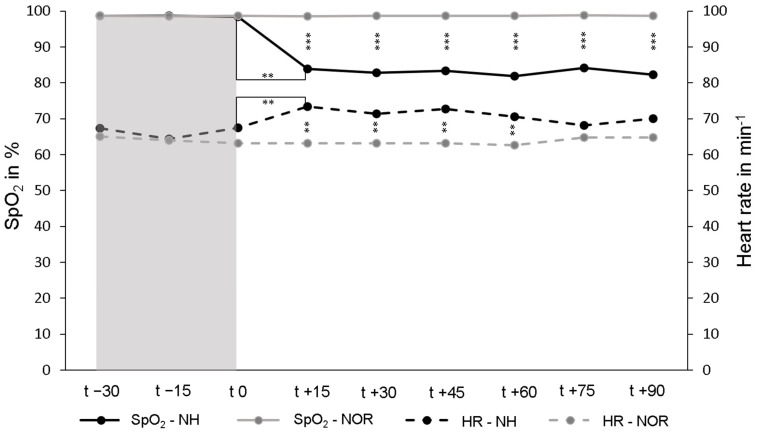
Peripheral oxygen saturation (SpO_2_, solid lines) and heart rate (HR, dashed lines) during the resting phases (t − 30–t 0, normoxia [NOR], gray area) and the subsequent normobaric hypoxia (NH) or continued NOR (t 0–t + 90, white area). The values shown are the mean values of the respective measurement times. ** *p* < 0.01; *** *p* < 0.001.

The mean SpO_2_ under NOR in the resting phase over all participants in the first 30 min was 98.7 ± 0.6%. Under continuing normoxia for another 90 min, SpO_2_ remained unchanged. Under NH, the average SpO_2_ decreased to 82.9 ± 4.2% and was significantly lower than under NOR (*p* < 0.001). To achieve a SpO_2_ value of 85–80%, an individual FiO_2_ value of between 14.5% and 10.5% was required for each participant. Under NH, the heart rate increased significantly to an average of 70.8 ± 10.1 min^−1^ and remained almost stable for the duration of NH. Under NOR the heart rate was on average 63.6 ± 9.6 min^−1^ and was significantly lower than under NH (*p* < 0.001) (Figure 1).

### 2.2. Effect 30 min Resting Phases under Normoxia on BDNF

**Figure 2:**
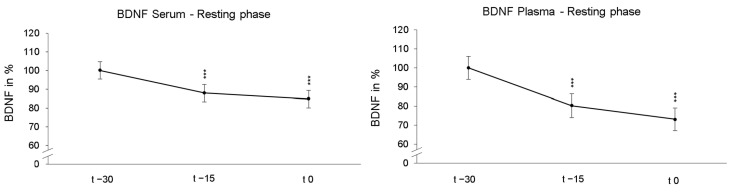
The presentation of the relative time course values in serum and plasma during the resting phases show the significant BDNF decline after 15 min (t − 15) and 30 min (t 0) under normoxic conditions. The initial group values were standardized for t − 30 to 100%. In addition, the standard errors are shown graphically. *** *p* < 0.001.

Participants first laid down in a supine position under NOR for 30 min (resting phases). As these resting phases were adjusted before the following 90 min of NH or NOR, each participant underwent the identical resting phase twice. First, we compared the reliability of BDNF measurements under passive NOR. The rmANOVA revealed a significant decrease in serum BDNF levels after 15 min (*p* < 0.001, η_p_^2^ = 0.616) and 30 min (*p* < 0.001, η_p_^2^ = 0.581) during resting phases. Similarly, the average plasma BDNF values also significantly decreased across participants after 15 min (*p* < 0.001, η_p_^2^ = 0.363) and 30 min (*p* < 0.001, η_p_^2^ = 0.362). The calculations of the individual resting phases showed no significant between-group effects. Figure 2 shows the time course of BDNF levels of all participants averaged over both resting phases and were normalized and related to the values obtained at time point t − 30.

### 2.3. The Effect of 90 min Normobaric Hypoxia on BDNF

**Figure 3:**
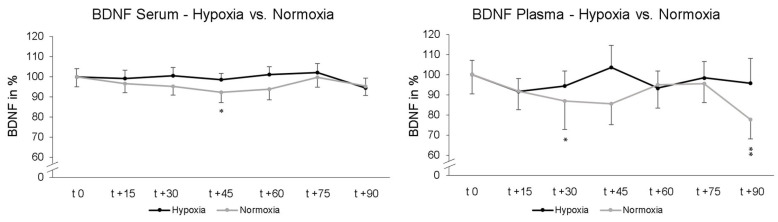
Time course of serum and plasma BDNF blood levels under hypoxic condition (black line) or while maintaining normoxic condition (grey line). Here, measurement time t 0 is the standardized relative initial value at 100%. Under maintaining normoxic condition there were significant decreases in serum BDNF 45 min (t 0–t + 45) and in plasma BDNF 30 min (t 0–t + 30) as well as 90 min (t 0–t + 90). Under hypoxic conditions, there was no significant decline in BDNF. Standard errors are also shown graphically. * *p* < 0.05, ** *p* < 0.01.

Two-way rmANOVA showed no significant interaction effect of “intervention x repeated measurement” upon serum BDNF levels after 90 min (*p* = 0.233; η_p_^2^ = 0.029). Post-hoc paired-sampled t-tests showed a significant decrease in serum BDNF levels after another 45 min NOR (t 0–t + 45; *p* = 0.018). Under NH no significant alteration in serum BDNF was found at any time. The data were normalized and are represented relative to the values at time point t 0. For plasma BNDF levels, two-way rmANOVA showed no significant interaction effect of “intervention x repeated measurement” after in total 90 min NH and NOR (*p* = 0.383; η_p_^2^ = 0.023). Post-hoc paired-sampled t-tests showed significant decline in BDNF levels under NOR after 30 min (t 0–t + 30; *p* = 0.040) and after 90 min (t 0–t + 90; *p* = 0.005). Post-hoc paired-sampled t-tests showed no significant alteration in BDNF plasma levels under NH at any time point (Figure 3). The data were normalized to the value at time point t 0 and represent relative data.

### 2.4. 24 h Follow-Up

**Figure 4:**
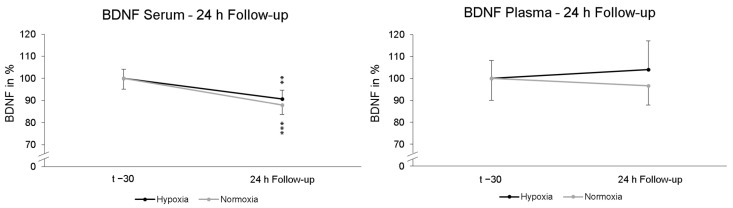
Serum and plasma BDNF levels at 24 h Follow-up relative to baseline (t − 30) and. In serum, the BDNF level was significantly reduced 24 h after NH (black line) and NOR (grey line). In plasma, there were no significant alterations in BDNF levels. Measurement time t − 30 is the standardized relative initial value with 100%. Additionally standard errors are shown graphically. ** *p* < 0.01, *** *p* < 0.001.

To find out whether a possible late effect on BDNF due to NH would occur, another serum and plasma sample was collected after 24 h. These data were compared to the first serum and plasma blood sample the day before (t − 30). No significant “intervention × Follow-up” interaction effect (*p* = 0.444; η_p_^2^ = 0.013) in serum BDNF was found. Post-hoc paired-sampled t-tests showed significant reductions in serum BDNF levels after NH (*p* = 0.004) and NOR (*p* < 0.001). In plasma, no significant interaction effect in BDNF levels was found for 24 h follow-up “intervention × Follow-up” (*p* = 0.684; η_p_^2^ = 0.004). Post-hoc paired-sampled *t*-tests also revealed no significant changes in plasma BDNF levels 24 h after NH (*p* = 0.339) and NOR (*p* = 0.924) (Figure 4).

### 2.5. Effect of Peripheral Venous Catheter vs. Butterfly Needle on BDNF

**Figure 5:**
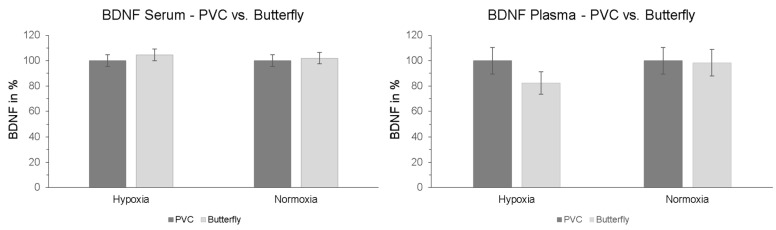
Serum and plasma BDNF levels after finishing hypoxic and normoxic conditions. Blood samples were taken simultaneously after 120 min (30 min resting phase + 90 min NH or NOR) via PVC (dark grey columns) and butterfly needle (contralateral arm, bright grey columns). Butterfly value is represented relative to PVC (initial value: 100%). PVC = peripheral venous catheter. Standard errors are also shown graphically.

Furthermore, we investigated whether the method of blood sample collection had a direct influence on measured BDNF levels. To determine whether there is a difference between the two types of sampling, another serum and plasma blood sample was collected via butterfly needle on the contralateral arm simultaneously with the blood sample from the PVC at the last measurement time point of the intervention (t + 90). The results showed no significant interaction effect “intervention × type of sampling” in serum (*p* = 0.529; η_p_^2^ = 0.009) or plasma (*p* = 0.183; η_p_^2^ = 0.041). Also, no significant effects of the type of sample collection were detected in post-hoc paired-sampled t-tests (Figure 5).

### 2.6. d2-R Test

Initially, an interaction effect “intervention x repeated measurement” was detected in the d2-R test via rmANOVA (*p* = 0.022; η_p_^2^ = 0.104). Post-hoc paired-sampled *t*-tests also revealed significant improvements in the d2-R test after both, NH (*p* = 0.001) and NOR (*p* < 0.001) (Table 1).

### 2.7. Digit Span Test

The rmANOVA did not show a significant interaction effect “intervention × repeated measurement” in forward (*p* = 0.216; η_p_^2^ = 0.032) and backward (*p* = 0.828; η_p_^2^ = 0.001) recall. Post-hoc paired-sampled *t*-tests showed no significant alterations in forward recall, neither after NH (*p* = 0.919) nor after NOR (*p* = 0.051). The post-hoc paired-sampled t-tests also revealed no effects in backward recall after NH (*p* = 0.514) or NOR (*p* = 0.266) (Table 1).

## 3. Materials and Methods

The present study was designed as a randomized, controlled cross-over study (registration number: DRKS00034492) investigating the effects of controlled hypoxia on BDNF and executive functions. It received approval from the ethics committee at the Otto-von-Guericke University Magdeburg (Germany, ethical approval number 54/19) and complies with the Declaration of Helsinki.

### 3.1. Participants

In total, 25 young healthy participants (15 female) with a mean age of 25.8 ± 3.3 years were examined. The average BMI of the test participants collective was 22.3 kg/m^2^. All participants either played no sport at all or were recreational athletes. The participants were not allowed to perform any moderate-to-severe physical activity for at least 24 h before the respective interventions. Exclusion criteria included respiratory diseases (e.g. bronchial asthma, COPD), cardiovascular/neurological/psychiatric diseases, drug/alcohol use in the 24 h preceding the examination, history of altitude sickness, a stay at altitude within the last 3 months or regular smoking habits. After being informed about the intention and the risks of the examinations, each participant signed an informed consent form, thus confirming their voluntary participation.

### 3.2. Experimental Design

**Figure 6:**
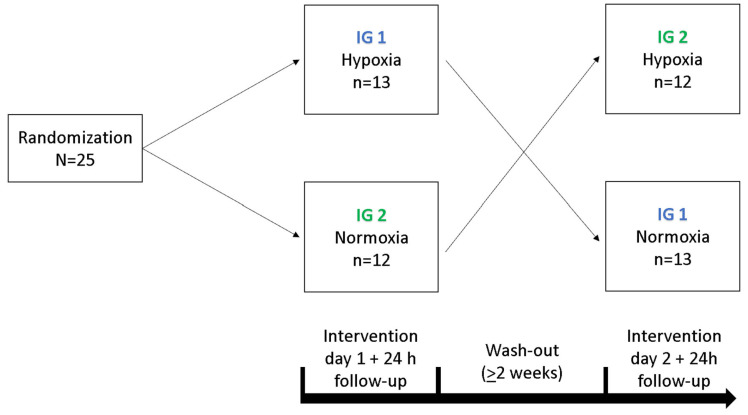
Illustration of the cross-over design for both intervention groups (IG 1, IG 2).

**Figure 7:**
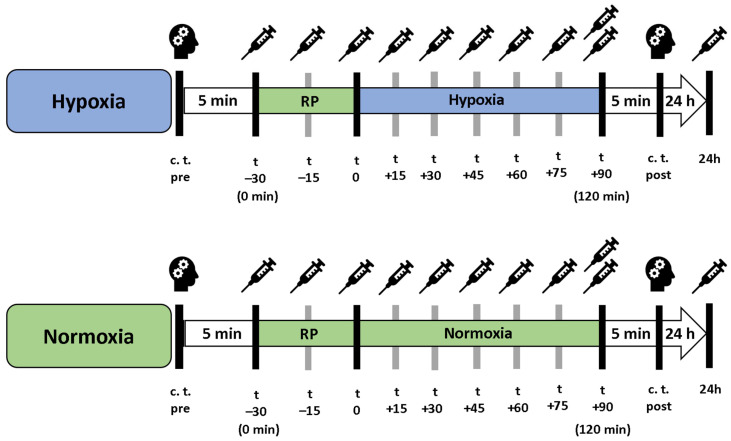
Procedure of the interventions under hypoxia (blue bar) and normoxic conditions (green bar). “Resting phase” and “Normoxia” are identical conditions. c. t. = cognitive testing, RP = 30 min preceded resting phase.

In a cross-over study design, each participant underwent a 90 min exposure under controlled NH and NOR, each after a preceding resting phase of 30 min under NOR. A delay period of two weeks occurred between the interventions, the order was randomized and counter-balanced (Figure 6). Before and after each intervention, cognitive tests were performed to assess short-term memory, working memory and attention performance. After the initial cognitive tests, each participant received a peripheral venous catheter (PVC) in the Vena mediana cubiti. Afterwards, participants remained in supine position for 120 min in total. During the first 30 min, all participants breathed room air (resting phase, NOR), followed by either a 90 min exposure under NH or maintaining NOR. During the entire intervention, serum and plasma samples were taken from the participants every 15 min via the PVC. At the last blood collection (after 120 min), another plasma and serum blood sample were taken from the contralateral arm via butterfly needle to determine whether the collection material had an influence on the measured BDNF level. After 24 h, another serum and plasma blood sample was collected (Figure 7). To reduce intraindividual circadian variations in BDNF levels, participants performed both interventions at the same time of day. If a hemolytic reaction occurred or blood samples were otherwise not evaluable, the participant was excluded from the respective calculation.

The NH was generated by using the hypoxia generator Everest Summit II (Hypoxico^®^, New York, NY, USA). A continuous positive airway pressure (CPAP)mask was used for this. During the NOR intervention, participants breathed room air without CPAP-mask. To monitor SpO_2_, a pulse oximetry device was fixed on the right index finger throughout the entire intervention. A target SpO_2_ of 85–80% was aimed for during the NH. In case of deviations from these values, SpO_2_ was corrected via the altitude setting of the hypoxia generator. In addition to SpO_2_, the heart rate was continuously measured.

### 3.3. Measurement of BDNF Levels

After blood collection, plasma samples were directly stored in iced water for 7 min. Subsequently, plasma samples were centrifuged at 2.000× *g* for 15 min. Serum samples were stored at room temperature for 37 min and then also centrifuged at 2.000× *g* for 15 min. After centrifugation, samples were directly aliquoted. Subsequently, the samples were frozen at −80 °C. Sandwich ELISAs were used for the determinations of quantification of BDNF (BDNF DuoSets; R&D Systems, Wiesbaden, Germany) as described previously [39]. This ELISA test kit detects mature BDNF protein and its uncleaved precursor pro-BDNF.

### 3.4. d2-R Test

The d2-R test is a well-established and validated test for determining individual attention skills. It was performed twice for each intervention, once before and once after finishing the interventions. In this test the letters “d” and “p” were each marked with one to four dashes. The participants were instructed to find and mark as many “d”s with two dashes as possible. All “d”s with more or less than two dashes and all “p”s should be ignored. In total, 14 rows with 57 letters each were presented (in total 798 letters). All 14 rows were processed one after the other without a break in between. The experimenter tracked the time and informed participants after 20 s to move on immediately to the next row. The evaluation of the d2-R test was performed according to the test manual of Brickenkamp et al. [43]. The individual attention performance was calculated by counting the total number of detected target letters minus the number of incorrectly marked letters.

### 3.5. Digit Span Test

The digit span test is a subtest of the Wechsler Adult Intelligence Scale and is designed to measure short-term memory and working memory performance [44]. During this test, a series of numbers was presented to the participants. The participants heard the series of numbers only once and were asked to reproduce them immediately thereafter (forward recall, short term memory). After finishing the first part, the second part was started straight away. In the second part of the test, participants had to repeat the series of numbers in reversed order (backward recall, working memory). The sequence length increased by one number with every second trial and new numbers were used for every trial.

### 3.6. Statistical Analysis

Statistical analysis of BDNF levels and cognition were performed with SPSS 26.0 (Inc./IBM, Armonk, NY, USA). The intervention effects for BDNF were tested using one-way and two-way repeated measures ANOVA (rmANOVA). To calculate the mean differences of the dependent samples, post-hoc paired-sampled *t*-tests were used. To determine a statistical correlation between two variables, *p*-value for significance was calculated. The significance level was set at *p* < 0.05 (*). If the value was *p* < 0.01 (**), it was considered highly significant, and if *p* < 0.001 (***), it was considered very highly significant. Values with a Z-score > 2.58 were considered as outliers and removed if not explainable [45]. Extreme values were also identified as such if a value was more than three times the interquartile range above the third or below the first quartile.

## 4. Discussion

Our data show that a passive resting phase of 30 min under NOR resulted in a significant reduction in serum and plasma BDNF levels with large effect sizes. To date, a large number of studies have shown that physical activity is associated with increased BDNF levels [2,21,46,47,48]. Studies investigating the short-term effects of physical inactivity on BDNF are still missing. Tarassova and colleagues used a 15 min resting phase before starting BDNF measurements and interventions [40]. However, in their study BDNF levels were not measured until the resting phase had ended. Therefore, in further studies, a standardized protocol for resting periods should be established before collecting serum or plasma blood samples for BDNF determination.

The nearly stable BDNF levels under passive NH compared to BDNF decline under NOR could result from physiological hemodynamic processes: previous studies showed a hypoxia-induced release of catecholamines, e.g., from the chromaffin cells in the adrenal medulla and cells in the glomus caroticus [49,50,51]. The catecholamines adrenaline and noradrenaline lead to an increase in heart rate, blood pressure and cardiac output [52]. This in turn increases the shear forces in blood vessels, which leads to increased activation of thrombocytes [53]. As platelets are the main store for BDNF in the periphery, more BDNF is also released when the platelets are activated [54]. There are also studies that have shown that an increase in blood lactate, e.g., during anaerobic physical activity, can lead to increased BDNF secretion [55,56]. In earlier studies, however, no increased lactate levels could be detected after passive controlled hypoxia [57,58]. For this reason, no lactate was determined in our study. However, the relatively stable BDNF levels under NH in our studies might be explained by the findings of Vermehren-Schmaedick et al. and Baker-Herman et al., who demonstrated increased BDNF levels in the brainstem and spinal cord after hypoxic interventions in rats and mice [25,59]. However, the underlying mechanisms are still largely unknown. Another finding here is that the type of blood collection system (peripheral venous catheter vs. butterfly needle) did not exert a significant effect on measured serum and plasma BDNF concentration. Thus, both systems are suitable for sample collection in further studies. In addition, there were no negative effects on executive functions caused by passive controlled hypoxia.

Controlled hypoxia is known for its low risks and potential short- and long-term benefits on physiological parameters (e. g., increased erythropoiesis, angiogenesis, antioxidant capacity, improved exercise performance) [26,27,60,61]. A recent review suggested that controlled hypoxia increases BDNF levels in the brain as well as in the blood of rodents [62]. However, the number of human studies investigating acute controlled hypoxia and its effects on BDNF is still very limited and the results are partly inconsistent. Here, we investigated the effect of controlled NH exposure and prior NOR on BDNF blood levels and cognitive performance in young, healthy adults at rest. By blood sampling intervals of 15 min during each of the 2 h interventions, we wanted to find out whether a BDNF time course under passive NH or NOR is detectable.

Currently, the number of human studies investigating BDNF blood levels in response to controlled hypoxia is limited. The results of our study are similar to those of Hubold et al. [38], who also showed no significant reduction in BDNF after 30 min of passive hypoxia compared to normoxia. However, in their study, in the follow-up (1 h after the intervention), BDNF levels were significantly higher after hypoxia compared to normoxia. Our analysis of the time course of BDNF levels showed that controlled hypoxic conditions and normoxic conditions both tend to lead to reduced BDNF levels, but this reduction was less pronounced in the hypoxia group. However, there was no significant between-group effect at any measurement time. A possible reason for this could be that each participant might need an individual reduction in SpO_2_ or hypoxia duration to maximize BDNF levels. Further regression analyses showed no gender-specific difference at any measurement time.

There are also studies reporting increased BDNF levels following a longer hypoxia exposure, e.g., after 72 h of continuous hypoxia [22]. In another study, in which BDNF was increased with a hypoxia duration of only 30 min, no predefined SpO_2_ but a fixed FiO_2_ was used [24]. In this study, a rigid FiO_2_ of 13.5% resulted in a strong decrease and fluctuations down to SpO_2_ of 94–63%. In order to limit such fluctuations in SpO_2_, we decided to use a predefined SpO_2_ instead of a predefined FiO_2_, as is usually the case.

Moreover, only a few previous studies investigated post-intervention follow-up of BDNF levels. Roeh and colleagues investigated serum BDNF levels 24 h and 72 h after marathon running. They found a stable BDNF after 24 h and significant reductions in serum BDNF was shown after 72 h [63]. Our study showed a significant reduction in BDNF levels after 24 h, both after NH and NOR.

Further studies could investigate whether a significant drop in BDNF level in physical inactivity would also occur when hypoxia was performed first and then followed by supine normoxic resting phase. With expanded knowledge about individual dosing and application, hypoxia application may have the potential to be an adequate treatment option to counteract cognitive age- or disease-related decline.

In summary, our data underline (i) the fundamental importance of standardized blood sampling protocols, as even a short period of physical inactivity had a significant impact on BDNF levels in serum and plasma. Furthermore, it was shown (ii) that controlled hypoxia has the potential to counteract the decline of BDNF in response to physical inactivity. In addition, (iii) our study shows partly consistent [38] and inconsistent [22,24,25,37] results in BDNF levels compared to previous studies. Therefore, further research on the potential influence of hypoxia on BDNF levels is needed.

Recommendations for a standard protocol for perspective BDNF investigations in humans:

1. Standardized protocol for blood collection (arrival, sitting position for 30 min, avoiding time deviations).
2. Standardized pre-analytic blood protocol (plasma: after blood collection 5 min blood sample in ice water, 15 min centrifugation at 2.000 g, aliquoting and storage at −80 °C; Serum: after blood collection 35 min blood sample storing at room temperature, 15 min centrifugation at 2.000 g, aliquoting and storage at −80 °C).
3. Strict patient/participant recommendations and instructions prior to blood collection (e.g., no intensive physical exercise interventions in the last 3 days prior to blood collection, no vigorous physical activities directly before blood collection)
4. Collection of potential influencing factors (e.g., smoking habits, potential use of drugs or alcohol, prior stays at altitude, diseases).

## 5. Limitations

An important limitation of this study is the small number of participants (N = 25). It is also conceivable that each participant requires individual SpO_2_ to achieve increased BDNF release. Despite exact adherence to the protocols, unintentional influence on the measurement results may have occurred, e.g., due to varying stress levels of the participants. Furthermore, the physiological responses (including respiratory and cardiovascular) to hypoxia differ between individuals, as confirmed also in the present study. Another limitation is that BDNF levels, especially in plasma, show a wide inter- and intraindividual spread of variance. In addition, it should be noted that the participants in our study required FiO_2_ between 14.5% and 10.5% to reach the predefined SpO_2_ of 85–80%. Thus, the individual workload and physiological response was divergent for each participant. Intraindividually, there were also fluctuations in SpO_2_ during the hypoxia exposure, so that in some cases FiO_2_ corrections of up to ±4.0% had to be made on the altitude air generator. Additionally, stress (sympathetic activation) during hypoxia could be another potential influencing factor. In animal studies, it has been shown that BDNF levels decrease when the animals have previously experienced stress periods [64,65,66]. Thus, in our studies, too-high stress levels due to hypoxia with SpO_2_ of 85–80% might also have been a reason for not increasing BDNF levels in serum or plasma significantly. It would therefore be advisable to also determine the blood cortisol levels for further examinations.

## 6. Conclusions

Our data show significant decreases in serum and plasma BDNF levels following a passive normoxic resting phase of 30 min. Continuation of the normoxic conditions led to significant additional reductions in BDNF levels in both serum and plasma. However, the exposure to controlled hypoxia counteracted this further decrease. Given the great potential of BDNF and controlled hypoxia for human health, the role of BDNF in the response to controlled hypoxia needs to be further elucidated. The definition of a uniform standard protocol (as suggested here) is therefore crucial for future studies.

## Figures and Tables

**Table 1:** Results of the cognitive tests, DST = Digit span test., AP = attentional performance.

	Hypoxia			Normoxia		
	pre	post	*p*	pre	post	*p*
d2-R-test–AP	208.36	218.08	0.001	203.44	221.84	<0.001
DST-forw. recall	11.44	11.48	0.919	10.92	11.60	0.051
DST-backw. recall	7.96	8.24	0.514	7.88	8.28	0.266

## Data Availability

Due to data protection regulations (ethical approval, DZNE requirements), the free publication of the data is not permitted. The presented data are available on request from the corresponding author.

## Abbreviations

BDNF	Brain-derived neurotrophic factor
CNS	Central nervous system
COPD	Chronic obstructive pulmonary disease
FiO_2_	Fraction of inspired oxygen
NH	Normobaric hypoxia
NOR	Normoxia
η_p_^2^	Partial eta squared
O_2_	Oxygen
paO_2_	Arterial oxygen Partial pressure
PVC	Peripheral venous catheter
rmANOVA	Analyses of variance with repeated measurements
SpO_2_	Peripheral oxygen saturation
